# Lessons learned in application driven imaging agent design for image-guided surgery

**DOI:** 10.1007/s00259-024-06791-x

**Published:** 2024-06-20

**Authors:** Tessa Buckle, Daphne D. D. Rietbergen, Linda de Wit -van der Veen, Margret Schottelius

**Affiliations:** 1 Interventional Molecular Imaging Laboratory, Leiden University Medical Center, Leiden, The Netherlands; 2 Section Nuclear Medicine, Department of Radiology, Leiden University Medical Center, Leiden, The Netherlands; 3 Department of Nuclear Medicine, The Netherlands Cancer Institute, Antoni van Leeuwenhoek Hospital, Amsterdam, The Netherlands; 4 Translational Radiopharmaceutical Sciences, Department of Nuclear Medicine and Department of Oncology, Centre Hospitalier Universitaire Vaudois (CHUV), University of Lausanne, Rue du Bugnon 25A, Agora, Lausanne, CH-1011 Switzerland; 5 Agora, pôle de recherche sur le cancer, Lausanne, Switzerland

**Keywords:** Image-guided surgery, Imaging agent development, Tracer development molecular design, Pharmacokinetics, Receptor targeting

## Abstract

To meet the growing demand for intraoperative molecular imaging, the development of compatible imaging agents plays a crucial role. Given the unique requirements of surgical applications compared to diagnostics and therapy, maximizing translational potential necessitates distinctive imaging agent designs. For effective surgical guidance, exogenous signatures are essential and are achievable through a diverse range of imaging labels such as (radio)isotopes, fluorescent dyes, or combinations thereof. To achieve optimal in vivo utility a balanced molecular design of the tracer as a whole is required, which ensures a harmonious effect of the imaging label with the affinity and specificity (e.g., pharmacokinetics) of a pharmacophore/targeting moiety. This review outlines common design strategies and the effects of refinements in the molecular imaging agent design on the agent’s pharmacological profile. This includes the optimization of affinity, pharmacokinetics (including serum binding and target mediated background), biological clearance route, the achievable signal intensity, and the effect of dosing hereon.

## Introduction

**Fig. 1:**
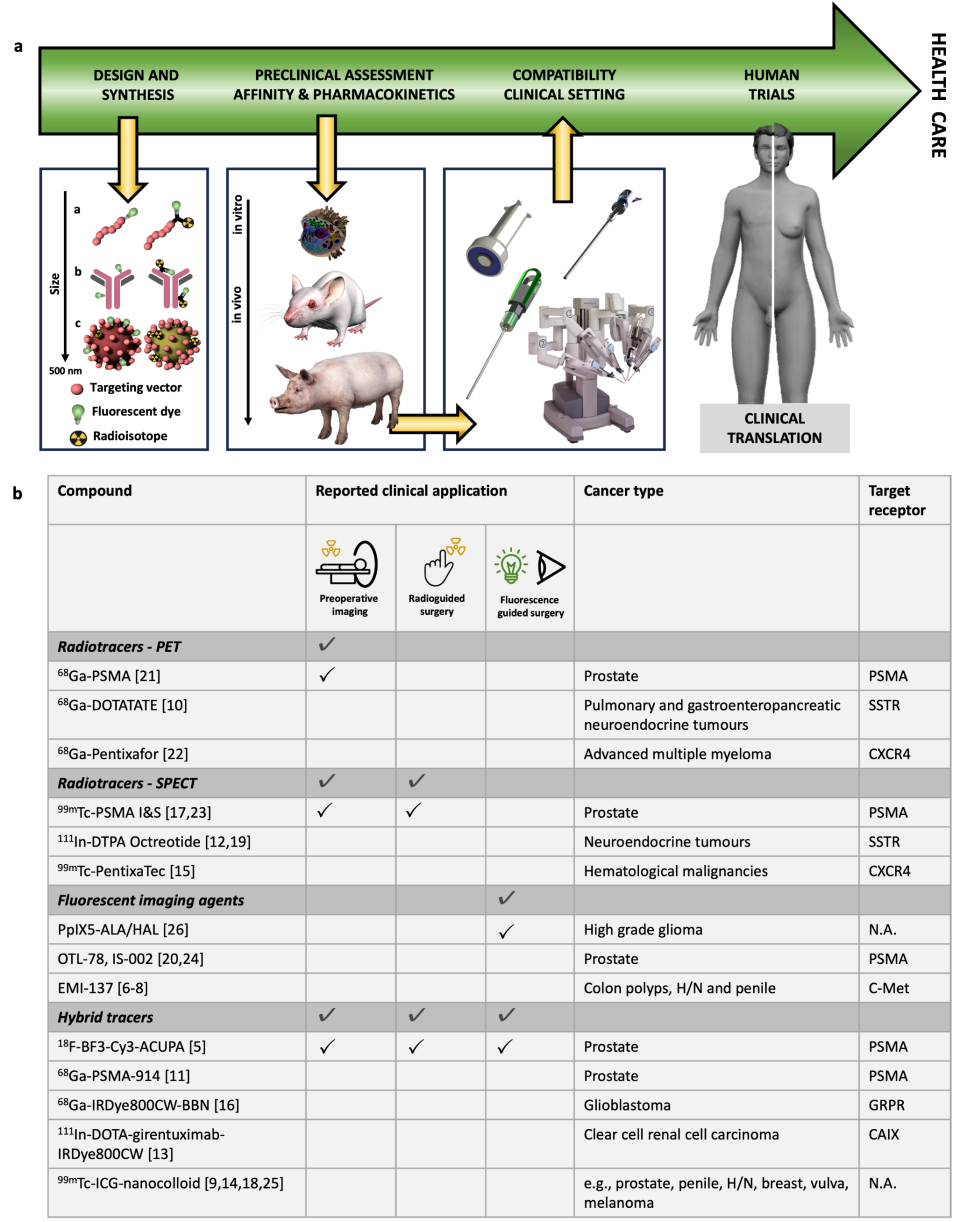
The translational process of novel tracers/imaging agents for image-guidance approaches. (**a**) Overview of the different steps in the translational process of novel agents for image-guided surgery applications, ranging from compound design and synthesis (with differences indicated between targeting vectors and imaging labels), preclinical assessment (in cells, mice and large animal models), compatibility with imaging equipment used in the clinical setting (open, laparoscopic and robotic surgery) to first-in-human clinical trials and ultimately application in clinical routine. (**b**) Examples of radiotracers for preoperative imaging, radiotracers for radioguided surgery, fluorescent imaging agents for intraoperative fluorescence-guided surgery and hybrid tracers for combined pre- and intraoperative imaging, based on their reported clinical applications

In the field of oncology, surgical resection of malignant tissue is the primary treatment option for many solid tumours, and incomplete resection has a major impact on subsequent treatments and patient survival. In recent years, there has been significant progress in the development of innovative imaging agents tailored for image-guidance during oncological surgeries to localize (suspected) tumour lesions, allow margin assessment with the aim to reduce residual cancer, or identify critical structures during the procedure [1, 2]. Various imaging agent designs that include a broad palette of different chelates (e.g., mas_3_, HBED-CC, DOTA, for complexation with respectively radioisotopes ^99m^Tc, ^68^Ga or ^111^In; [3, 4]), fluorescent dyes with different fluorescent emissions (e.g., FITC, Cy5, IRDye800CW, Indocyanine green (ICG); [4]) or a combination of imaging labels (so called bi-modal or hybrid tracers; [2]) have undergone the process of clinical translation (Fig. 1A), and have ultimately been implemented in the treatment of various different tumour types (Fig. 1B; [5–26]). Based on these success stories intraoperative molecular imaging is rapidly emerging as a unique new theranostic strategy [27, 28].

The transition from exclusive preoperative diagnostics to the facilitation of image-guided surgery has been made possible via of use of beta- and gamma-emitting tracers in different applications, and the possibility of combination of these signals with other signals when hybrid tracer designs are being pursued. A prime example of expansion of the use of radiotracers is their use in intraoperative guidance, which has been made possible through the availability of hand-held detectors. The most popular are gamma probes or portable cameras that enable in vivo detection of γ-emissions from radionuclides originally designed for SPECT, but ß-probes that allow intraoperative detection of PET tracers and drop-in gamma probes that allow intraoperative robotic SPECT are also reported [3, 29, 30]. This so-called “radioguided surgery” (RGS) concept has been pioneered in sentinel lymph node biopsy, a procedure that is aimed at identifying micrometastasis in tumour draining lymph nodes via the lymphatic drainage of radiolabelled colloids [31, 32]. RGS nowadays also encompasses receptor-targeted applications using tracers that specifically bind to malignant cells or components of the tumour microenvironment such as somatostatin receptors (SSTR, [12, 19]), prostate specific membrane antigen (PSMA, [17, 33]), chemokine receptor 4 (CXCR4, [15, 22]), anti-carcinogenic antigen (CEA, [34, 35]), and carbonic anhydrase IX (CAIX. [36]). And while to date only explored for diagnostic and therapeutic purposes, fibroblast activation protein (FAP) could potentially also serve as an interesting target for RGS [37].

Similar to radioligand therapy (RLT), modern receptor-targeted surgical applications rely on “theranostic companion tracers”, i.e. pairs of dedicated tracers labelled with different radionuclides (e.g. ^68^Ga for diagnostic PET and ^111^In/^99m^Tc for RGS). Examples of such tracer pairs have been reported for pre/intraoperative targeting of SSTR [10], the chemokine receptor 4 (CXCR4 [15, 22]), and PSMA [33]. This recent use of interventional molecular imaging (IMI) concepts is essentially an extension of a field originally poised as radioimmunoguided surgery [38, 39]. Overall, this strategy opens unique new possibilities for consecutive preoperative imaging and intraoperative detection (Fig. 1B), but at the same time increases the demand for optimal performance in two distinct clinical settings.

**Fig. 2:**
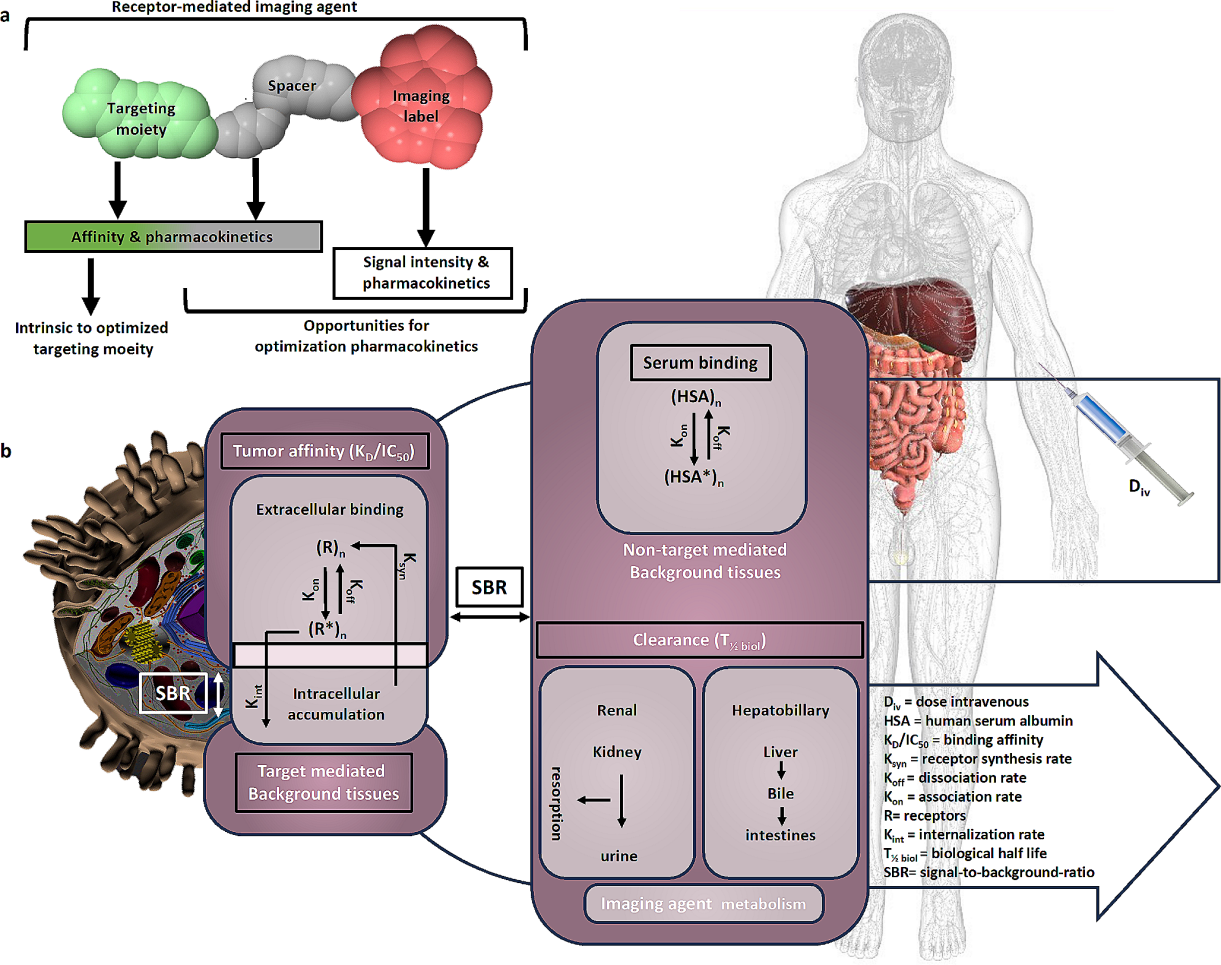
Imaging agent design and pharmacokinetic compartment model for receptor-targeted imaging agents. (**a**) General composition of a receptor targeted imaging agent that is composed out of a pharmacophore (in green), a spacer molecule (grey) and an imaging label (red), and the opportunities for imaging agent optimization. (**b**) Generic pharmacokinetic compartment model with key features of which tumour affinity, serum binding, the signal-to-background ratio (SBR) and clearance can be influenced via the imaging agent design. Anatomical image created using Biodigital.com

Furthermore, the increasing demand for precision-guidance that facilitates decision making during surgical procedures has necessitated the inclusion of guidance strategies that complement RGS. In this context, fluorescence plays an increasingly prominent role as a commentary technique [3]. Compared to the acoustic readout provided during radioguidance, fluorescent emissions facilitate real-time visual lesion identification [4]. On the other hand, radioguidance is based on sensitive whole tissue penetration of the signal of the radioisotope while fluorescence imaging is limited by tissue attenuation and sensitivity [2, 40]. Fluorescence guidance can either be implemented by the use of free fluorescent dyes such as fluorescein, ICG or PpIX (5-ALA/HAL) [41] or dyes conjugated to targeting vectors (fluorescent imaging agents and hybrid tracers; Fig. 1b, [1, 2]), . Depending on the specific requirements of the intended procedure and the available instrumentation (e.g., fluorescence endoscope, Firefly system in Da Vinci surgical robot setup, [42, 43]), dyes with a fluorescence emission ranging from the visible to the near-infrared (II) region can be selected [1, 4, 44]. More specifically, fluorescence signals do not penetrate beyond 1 cm through tissue, with real-time working-distance even being < 0.5 mm [40, 45]. In its earliest phase, imaging agent development is principally focused on stably integrating a radionuclide into a targeted biovector. Based on the inherently and reliably quantifiable signal, biodistribution and clearance can be assessed both in vivo and ex vivo [44, 46]. Today’s efforts tend to focus on detailed imaging agent optimization via small alterations in the chemical design e.g., tailoring of the chelate and/or the linker connecting the chelate and pharmacophore/targeting moiety [47, 48]. Even minor chemical modifications in the composition of the imaging label have been shown to have substantial effects of fundamental chemistry/biological relationship that ultimately drives the imaging agent’s pharmacokinetic profile. Herein the size of the targeting vector will be leading in the severity of the pharmacological impact (Fig. 1a, left image). For instance, this effect will be less pronounced for antibodies but more evident for peptides/small molecules.

For the latter the effect on affinity and pharmacokinetics is even more true for the integration of fluorescent dyes into an existing imaging agent design. Their influence on the general imaging agent characteristics is known to be substantial [46, 47, 49, 50], and especially so since development of fluorescent imaging agents often is not focused on minimizing this effect but adheres to generic design strategies that incorporate standard commercially available dyes such as IRDye-800CW. Moreover, the ability to optimize the in vivo utility of fluorescence-only imaging agents is hampered by the inability to quantitatively (and noninvasively) assess biodistribution and pharmacokinetics [2].

Generally, when looking at the currently available probes for receptor-targeted IMI applications in surgery, it becomes clear that a specific subset of features directly impacts the utility imaging agent towards impacting the clinical decision making, which is in turn determined by sensitivity, specificity, clearance, non-specific background and ease of detection [47]. There are, however, means to guide chemical designs such as to control these features, e.g. to strike a balance between systemic exposure and background signals [51] for specific surgical requirements. This review summarizes opportunities for optimization of the pharmacokinetics (Fig. 2) of peptide/small molecule-based imaging agents for IMI by tailoring chemical characteristics such as receptor affinity, plasma protein binding, general pharmacokinetics, the biological clearance route and ultimately signal intensity in the target tissue.

## Refinements in the molecular imaging agent design

### Strategies for optimization of affinity

Affinity (in the low nanomolar range; K_D_/IC_50_) of a receptor targeted imaging agent (Fig. 2a) is certainly one of the key parameters for efficient targeting, as is efficient internalization (K_int_, Fig. 2b) and subsequent detectability of the target tissue. Affinity is primarily driven by the selection of a pharmacophore that is optimized for its interaction with the receptor binding pocket. This has been broadly exemplified by the progressive optimization of e.g., small EuK inhibitors (PSMA-targeting) or cyclic peptides (SSTR targeting) [47, 52].

**Fig. 3:**
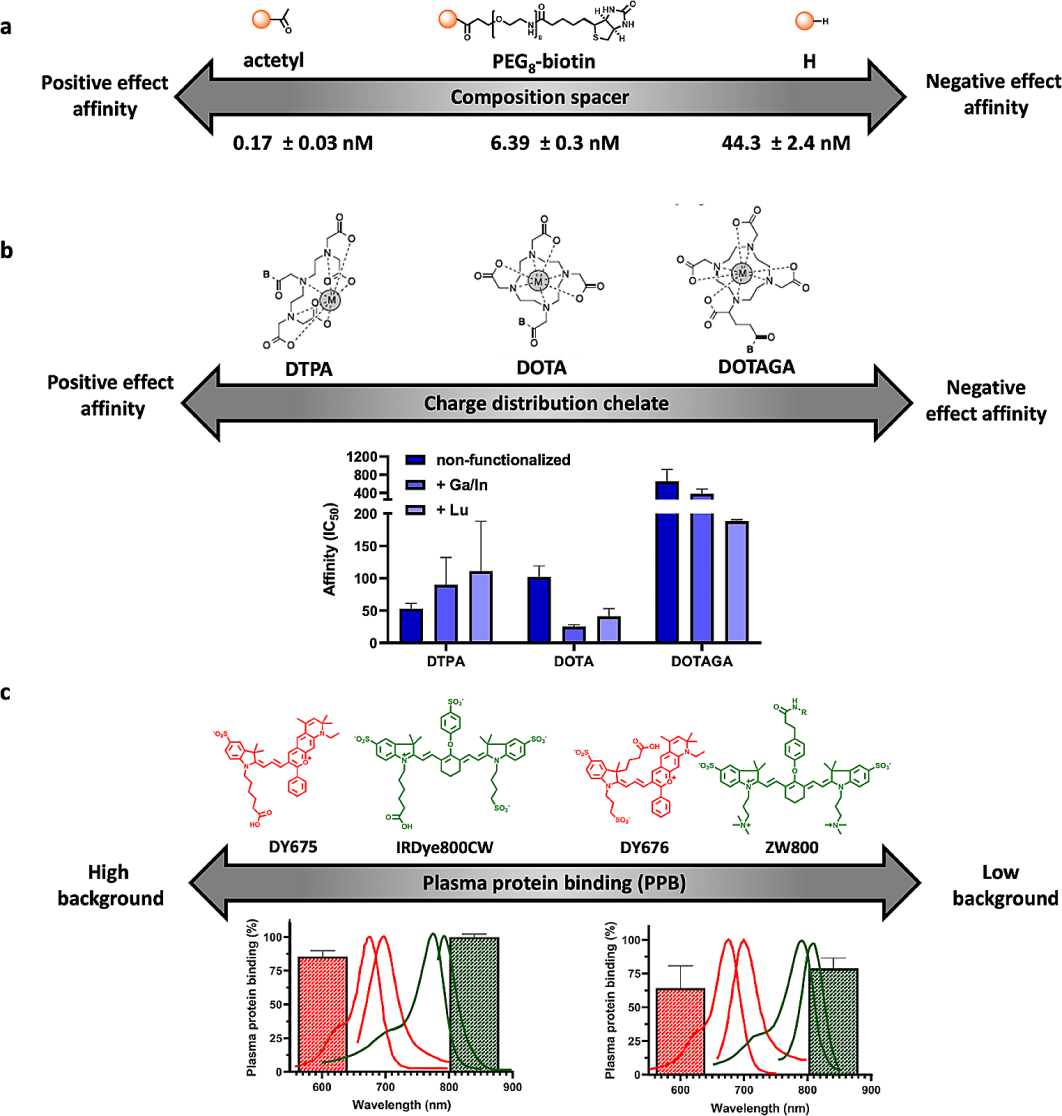
Strategies for optimization of affinity and plasma protein binding of receptor targeted imaging agents. Examples of the effect of (**a**) spacer composition on imaging agent affinity (PSMA, values obtained from [56]; peptide in orange), and (**b**) the use of different chelates on tracer affinity (CXCR4, values obtained from [48]). (c) Examples of dye asymmetric cyanine (in red) and typical near infrared (NIR, in green) dyes with their corresponding emission and percentage of PPB (values obtained from [65, 66]

Attachment of a substituent to a pharmacophore, however, may, depending on its position in the targeting moiety, interfere with the interaction of the ligand with the receptor binding pocket. This effect becomes increasingly prominent when relatively small pharmacophores are combined with relatively large and structurally rigid imaging labels. Such influences, however, may be minimized by e.g., introduction of spacers between the pharmacophore and the imaging label, and/or structural optimization of the spacer and the imaging label itself (Fig. 3a).

To mitigate the influence of steric hindrance, it is common to use spacers of different lengths to try and optimize the orientation of the imaging label with regard to the pharmacophore. This strategy has been successfully explored for i.e., for the gastrin releasing peptide receptor (GRPR [53, 54]), PSMA [47, 55–57], human epidermal growth factor 2 (HER2 [58]) and the folate receptor α [59]. Alternatively, the spacer can be used to promote complementary interactions within the binding pocket, essentially making it an integral part of the pharmacophore. A prime example hereof is the spacer optimization achieved in PSMA-radiotracers [60]. This performance-enhancing strategy could even be expanded towards the use of tailored fluorescent dyes that serve as the spacer [61].

Electrostatic interactions induced by the imaging label are also known to affect binding affinity and kinetics [46, 47]. The complex geometry and charges of different radiometal-chelates were shown to exert a major influence on receptor affinity for some compound classes (Fig. 3b [48, 62]). The same is true for fluorescent dyes [50, 61, 63, 64]. Hence, careful consideration of charge and charge distribution within the imaging label is another crucial factor to be considered in the optimization of affinity.

### Plasma protein binding

**Fig. 4:**
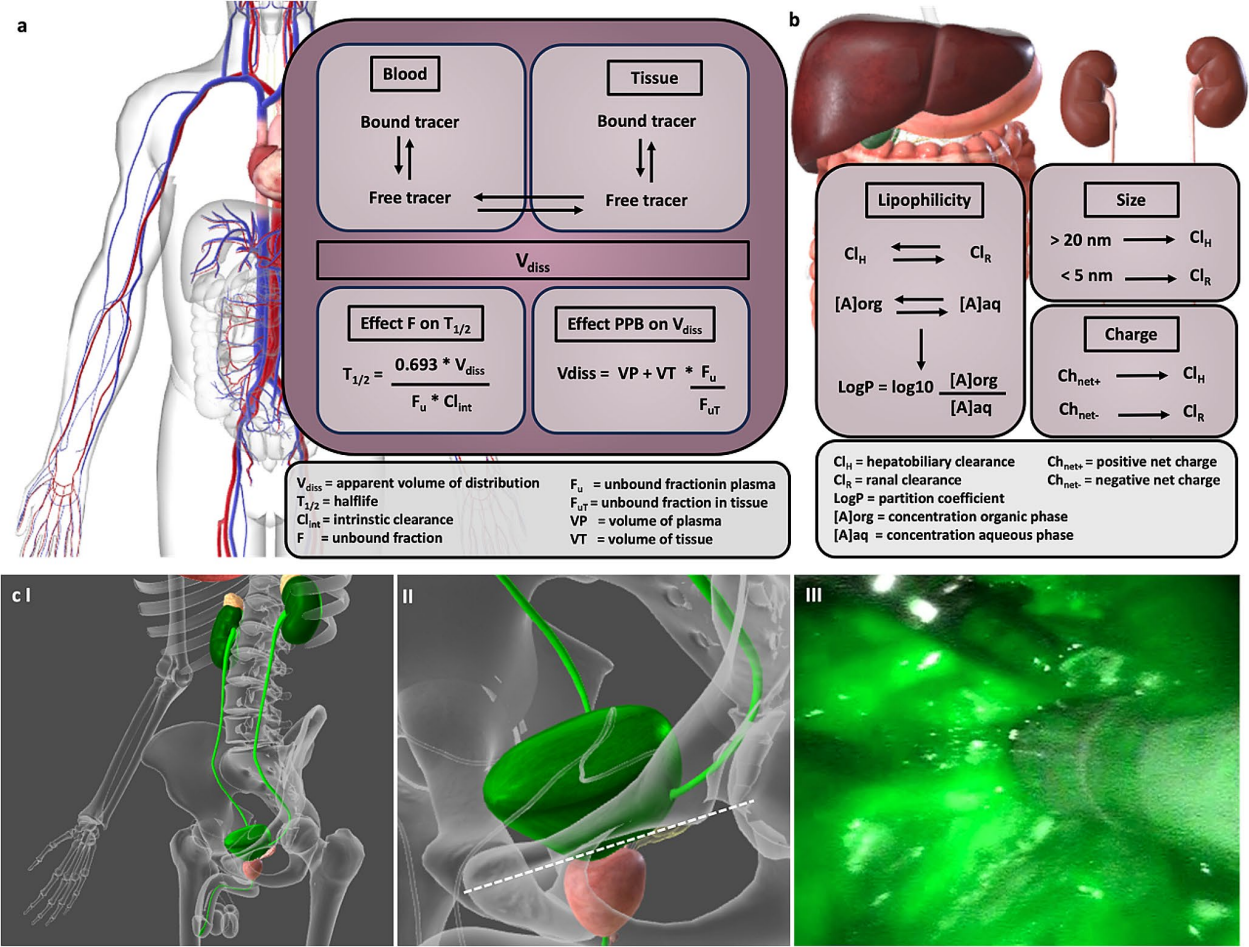
Effects of imaging agent characteristics on distribution and clearance, and clinical example of the effect of imaging agent clearance in image-guided surgery applications. (**a**) Model of imaging agent diffusion between the blood and tissue, the effect of the unbound imaging agent fraction on the agent halflife and plasma protein binding (PPB). Mathematical equations (obtained from [67]) highlight the underlying pharmacological mechanisms underline the fundamental chemistry/biological relationship that ultimately drive the imaging agents pharmacokinetic profile. (**b**) Effects of lipophilicity, particle size and net charge on imaging agent clearance [72]. (**c**) Clinical example the effect of renal clearance; I schematic representation of the renal clearance pathway, II dislocation prostate from tracer containing bladder during prostatectomy, III contamination with fluorescent dye-containing urine in the surgical field after prostatectomy in a prostate cancer patient. Anatomical images created using Biodigital.com

The in vivo performance of targeted imaging agents is also largely influenced by their affinity to serum proteins such as albumins (usually referred to as plasma protein binding (PPB) [67]). The equilibrium between serum-bound and free imaging agents determines the unbound fraction of imaging agent (F) that is available for receptor targeting within the volume of distribution (V_diss_ = (volume of plasma (VP) + volume of tissue (VT)) * (unbound fraction in plasma (F_u_) /unbound fraction in tissue (F_uT_); Fig. 4a). PPB largely determines the blood-retention, and as such, the biological half-life (T_1/2biol_ = (0.693 * V_diss_)/(F_u_ * intrinsic clearance (Cl_int_)) of an imaging agent and directly affects both the tumour uptake as well as nonspecific background retention (Figs. 2b and 4a [63, 67]). While long circulation times help increase target specific signal intensities by prolonged delivery of the imaging agent, they can also have a negative influence on signal-to-background ratio (SBR = specific signal – nonspecific background signal in the surrounding tissue), Fig. 2b [66]).

Prolonged presence of PPB induced high imaging agent concentrations in blood has been observed for a variety of radiotracers such as [^99m^Tc]PSMA-I&S [23] and ligands specifically modified with plasma-protein-binding moieties [68]. As fluorescent dyes such as indocyanine green (ICG) are known to exhibit high PPB, it is not surprising that dye structures can enhance the PPB of fluorescently labelled imaging agents (Fig. 3c, [65, 69–71]). Conversely, the incorporation of an increasing number of charged groups, as exemplified in asymmetric cyanine dyes, results in a progressive reduction of PPB [65]. A similar effect is seen for near infrared (NIR) dyes (Fig. 3c, in green [66, 69, 71]).

### Biological clearance route

Although not often addressed during preclinical or clinical evaluation, the clearance route of an imaging agent may largely affect its clinical applicability in IMI (Fig. 4b/c). For instance, in the context of urological surgery, renal clearance of fluorescent imaging agents can result in contamination of the surgical field with fluorescent urine (Fig. 4cIII). In the clinic such contamination has been reported to hinder intraoperative assessment of the basal margin of the prostate [24], but this also applies to visualization of fluorescence-containing lymph nodes located close to the prostate. Some suggest a strategy towards overcoming this limitation is to reduce the imaging agent content in the urine, either through increasing the hepatobiliary excretion or by matching the timepoint of imaging to the excretion window (dose interval prolongation; [24, 47]). Conversely, hepatobiliary clearance can hamper detection of hepatic lesions or lesions in the intestines. As such, the location of the target lesions can also be a factor that weighs in when designing a suitable imaging agent for a given application.

Generally, the molecular size and the sum of all structural components of a receptor targeted imaging agents (Figs. 2 and 4b) co-define its predominant excretion pathway (renal or hepatobiliary). Relatively small (< 5 nm), charged, and/or hydrophilic imaging agents tend to be renally excreted [69, 72]. Their precise structure, net charge [73] and charge distribution will determine the glomerular filtration and tubular reabsorption in the kidney [74]. These features in turn influence kidney uptake and retention [75]. Larger (> 20 nm) and/or more lipophilic imaging agents tend to follow the hepatobiliary clearance route [72], resulting in excretion into the bile and subsequently the intestines (Fig. 4b). A driving force for hepatic clearance is the above-mentioned PPB.

**Fig. 5:**
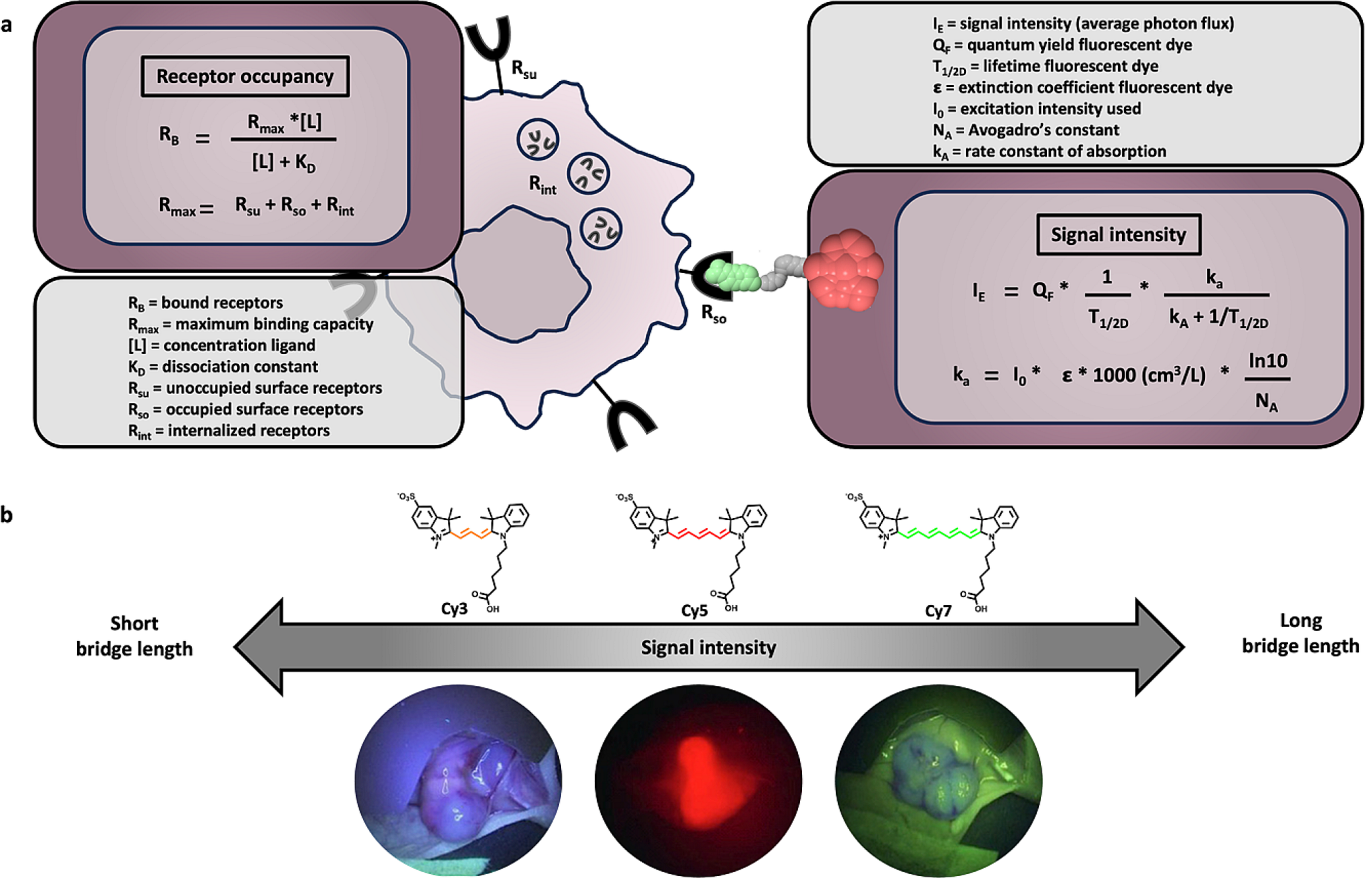
Receptor occupancy and signal intensity of a fluorescent label. **a**) Model of receptor occupancy and signal intensity of a fluorescent label. Mathematical equations. (adapted from [81, 82]) highlight the underlying pharmacological and optical mechanisms that describe the fundamental relationship between receptor availability and signal intensity. **b**) effect of variation bridge length in the dye component on signal intensity and in vivo tumour visualization (ανβ3 [76])

Furthermore, the composition of the fluorescent dye can be exploited to alter the biodistribution (Fig. 5c). Strategic placement of (charged) endgroups can be used to tailor imaging agent performance [50, 61, 76] as well as the route of excretion [49]. Assessment of a imaging agent matrix with varying endgroups has been shown to allow the evaluation of an particular variation and allow the selection of the most appropriate imaging agent derivative for the intended application [50, 61]. Current literature seems to indicate that each targeting vector requires it unique own optimization. While there are efforts ongoing to see if general trends can be observed, generic guidelines have not yet been reported.

Reduction of the dye lipophilicity (LogP = log10 * (concentration [A] in organic phase/[A] in aqueous phase); Fig. 4b) can positively affect tumour-to-background ratios, whereas increasing the number of charged moieties enhances renal uptake [46, 47, 50, 69, 77]. Interestingly, these effects can vary between targeting moieties, and different optimization approaches might therefore be needed for individual imaging agent molecules [46, 47, 50].

In some instances, it may be impossible to modify the route of excretion of a given imaging agent. In these cases, an alternative strategy consists in enhancing the excretion rate. Excretion rates are known to range from several minutes to several hours after administration for small molecules but may last up to several days after administration for antibody-based agents [1, 7, 78, 79]. Acceleration of the excretion rate can for instance be achieved by co-administration of diuretics. Longer time intervals between imaging agent administration and surgery also help to minimize background signals but may also be considered unfavourable for clinical logistics and do require highly stable receptor-target binding without significant dissociation or metabolization [80].

### Signal intensity

The surgeon’s ability to base his/her decision making on the imaging agent accumulation in a lesion depends for a large part on the signal intensity and SBR provided by the individual compound. Signal intensity (I_E_) is a function of the emission and absorption characteristics of the imaging label. For fluorescent imaging labels these include the quantum yield (Q_F_) and fluorescence lifetime (T_1/2D_) and the rate constant of absorption (k_A_) of the incorporated fluorescent dye (Fig. 5a). For receptor-targeted approaches I_E_ is highly influenced by the total number of occupied receptors (R_B,_ [81]) at a specific imaging agent concentration ([L]; Fig. 5a) and the K_d_ of the imaging agent used (R_B_ = (maximum binding capacity (R_max_) * [L])/([L] * K_d_)), and the detectability and intensity of the signal emitted by the imaging label (Fig. 5a [82]), . R_max_ (sum of the number of unoccupied surface receptors (R_SU_), occupied surface receptors (R_SO_) and internalized receptors (R_int_)) is determined by receptor kinetics ( [83, 84]; Fig. 2) and is inherent to the specific target receptor and tumour cell types and, in conjunction with the (heterogeneity of) receptor overexpression, they represent a translational challenge.

Detectability, in turn, also relies on the ready availability of sensitive detector instrumentation, and the imaging label must ideally be adapted to optimal detection by the respective hardware. Consequently, currently used imaging agents for intraoperative guidance are primarily labelled with ^99m^Tc (gamma-probe or portable gamma camera; 140 keV), ICG (near infrared fluorescence camera; l_ex_ = 800, l_em_ = 820 nm), PpIX (photodynamic diagnostics; l_ex_ = 488, l_em_ = 640 nm), and Fluorescein (photodynamic diagnostics; l_ex_ = 488, l_em_ = 515 nm), and ongoing imaging agent design revolves around the incorporation of these imaging labels. Efforts have also been made to enable direct detection of diagnostic PET-tracers (^68^Ga- or ^18^F-labelled) in the OR using either Cerenkov Luminescence or PET/CT specimen imaging devices, or beta-probes [85, 86] but these techniques did not find their way into clinical practice mainly due to the high noise-levels associated with scattered gamma-rays (e.g., limited signal detectability and contrast).

In patients, the biological complexity and heterogeneity of receptor expression in individual tumor lesions may result in relatively low signal intensities and subsequent low SBR for receptor-targeted imaging agents. This, of course, greatly influences and complicates surgical decision making [87]. This real-life finding seems to contradict with the promise offered by studies in preclinical, but highly artificial, models. It is highly relevant to consider the differences between preclinical models, often mice, and humans (physical size (20–40 gram vs. 60–80 kg bodyweight) and heart rate (500–700 vs. 60–100 beats/min), rate of excretion 1–2 mL vs. 800–2000 mL urine/day) and metabolism (5.3*10^5^ vs. 31.3*10^5^ kJ/kg **) [88, 89] and rate of tumour growth (0.016 vs. 0.08 mm^3^/day; [90, 91]).

**Fig. 6:**
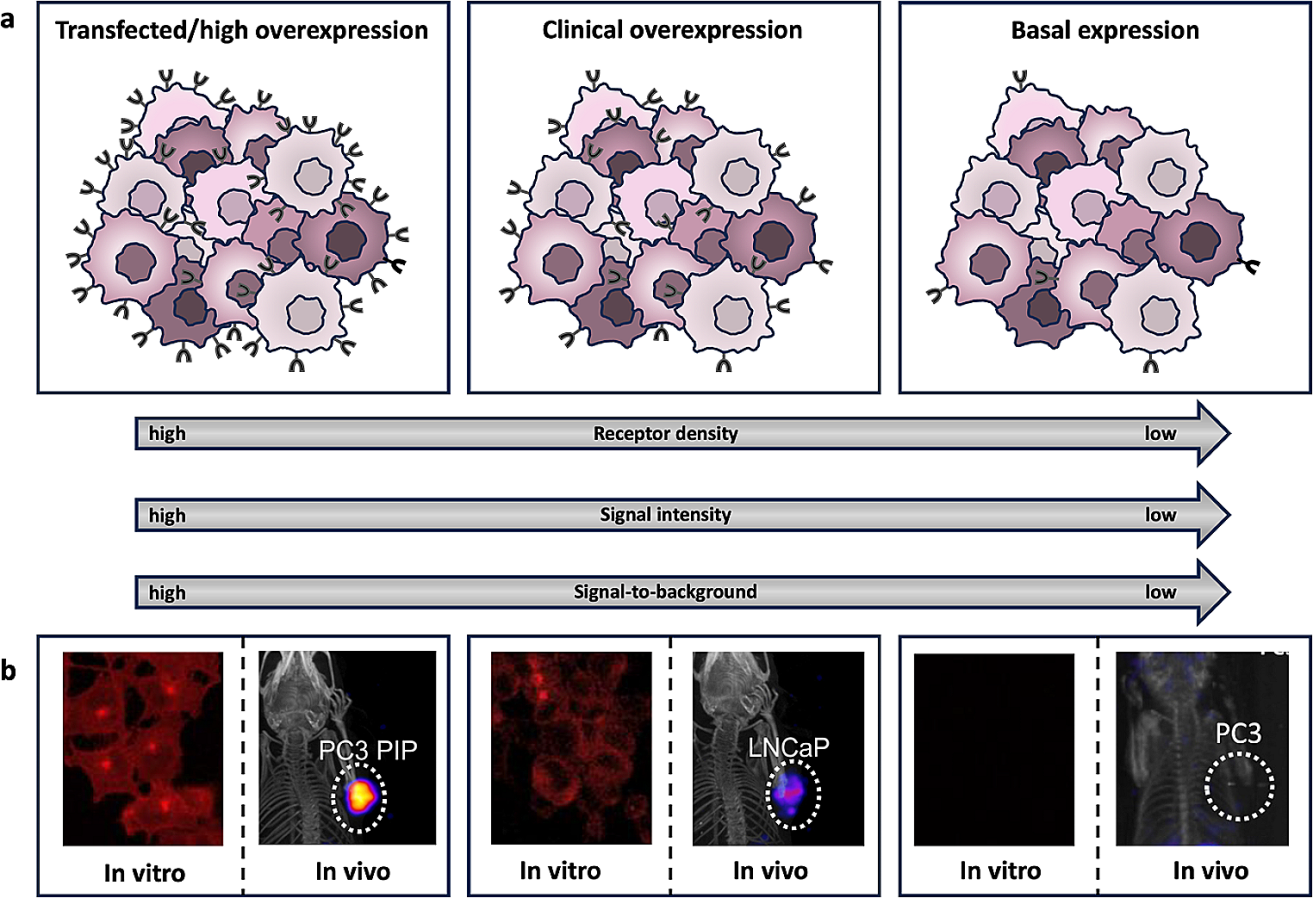
Influence receptor expression on tumour visualization. (**a**) schematic representation of the relation between receptor overexpression/receptor density, signal intensity and the signal-to-background ratio. (**b**) Examples of cellular uptake (in vitro) and tumour uptake (in vivo) for respectively IRDye700DX-PSMA (in vitro, all cell lines; [92] ), ^117^Lu-PSMA-617 (in vivo, PC3 PIP and LNCaP; [93]) and ^25^I-PSMA-7 (in vivo, PC3; [96]) showing differences in uptake with different PSMA expression levels

Also, the choice of cell line for in vitro and/or in vivo assessment directly influences the outcome of experiments (Fig. 6). As an example, high PSMA receptor expressing transfected cell lines such as PC3 PIP [92, 93] merely help validate targeting efficacy in the most ideal situation. Human derived PSMA expressing cancer cell lines such as LNCaP [92, 93] display more “natural” expression levels (Fig. 6) and can ultimately provide a more realistic signal intensity. Still, it has to be acknowledged that the physiological distribution of target-receptors can be quite different among species, complicating translation of imaging agent pharmacokinetics. PSMA for example, is not expressed to any significant degree in prostates of mice and monkeys [94], while in men and pigs this protein is present in normal prostate tissue and overexpressed in human prostate cancer [61, 95].

### Effect dosing on signal intensity

A clear difference between radiotracers and fluorescent imaging agents is that the first -independent of the target pursued- tend to follow the micro-dosing principle (*≤* 100 µg tracer or ≤ 30 nanomoles for protein products; [97–100]. During the design and implementation of radiotracers, high molar/specific activity (= high amount of radioactivity per mole or gram of compound in respectively Bq/mol or Bq/g [101, 102]), is preferred. Hereby the minimization of non-radiolabelled substituents in the administered formulation helps avoid receptor-saturation and selfblocking effects [103].

Because of light attenuation by tissue, during surgery the sensitivity of fluorescence detection is substantially lower than that of radioguidance [45, 99]. This feature increases the chance of false negative findings. As the administered dose of a fluorescent agent is related to the fluorescence intensity, this lack of sensitivity can -in part- be compensated for by increasing the administered dose [24, 104]. In addition, fluorescent imaging agents used in clinical trials tend to be dosed in the therapeutic window of the targeting agent used (e.g., EMI-137 (0.13 mg/kg [7]), OTL38 (0.025 mg/kg [105]), IS-002 (0.025 mg/kg [20]). This actually entails a formulation strategy that is quite the opposite of pursuing high molar/specific activity. As a result, fluorescence approaches are prone to receptor saturation and self-blocking effects. Increased dosing also brings other risks. For example, it enhances the risk of toxicological effects [102], which is a feature that substantially complicates translation. High dose regimens studies with fluorescent folate- and PSMA-targeted imaging agents report high background values, meaning higher background/off-target staining [20, 24, 105]. This increase in background promotes false-positive findings (up to 30%) with increasing dose, which correlates to unnecessary excision of non-diseased tissue and tends to reduce the SBR [24]. In turn, a low SBR complicates lesion identification. Furthermore, dose escalation has been shown to result in and thus a lower discriminatory ability for these agents for both locoregional and/or residual disease detection and identification of (metastatic) lymph nodes.

Hybrid tracers provide a means to employ the superior radiotracer sensitivity to reduce the amount of fluorescence needed and with that reduce the injected dose [99, 106]. For example, clinical use of ICG-^99m^Tc-nanocolloid (micro-dosing at ± 10 nmol HSA) shows effective fluorescence-based identification of the SN [9, 77]. In analogy to that, 100 µg of hybrid PSMA targeting tracer (^99m^Tc-EuK-(SO_3_)Cy5-mas_3_ (*h*PSMA), peptide; [93]) allowed for fluorescence imaging of basal-PSMA expression in a porcine model. This indicates that in dose optimization studies, large animal model evaluations could be used to bridge the gap between the relatively high dose per kg bodyweight used in preclinical studies and the pursuit of micro-dosing in clinical trials [7, 47, 106]. Such dose reduction efforts are supported by the constant sensitivity improvements of camera systems [85, 107] and additional AI and augmented reality-based guidance opportunities [108, 109]. The above-mentioned dose optimization is a practical component for the alignment of (radio)chemical developments with clinical requirements.

It must be said that while tracers such as ^99m^Tc-PSMA [110] and ICG-^99m^Tc-nanocolloid have been shown to provide patient benefit [18], this evidence has not yet been provided for fluorescent targeted imaging agents. At the moment there is a demand for high-level clinical evidence in the form of improved patient outcomes [18, 110] to indicate which strategies work in real-life patient care and which ones don’t. In addition, Delphi initiatives that focus on establishing the clinical demand in the form of e.g. timing, surgical readout [80] and preferred trial protocols are thought to help to substantially increase the translational success rate.

### Refinement of imaging label and signal intensity

An increase is signal intensity can also be achieved, either via the introduction of multiple signalling units in a single pharmacophore or by the optimization of the signal intensities in individual labels. The first strategy tends to conflict with the optimization of the affinity, because the potential for steric hindrance increases when multiple imaging labels are introduced [111]. Of note, in the case of fluorescent dyes, placement of multiple units within close vicinity of each other often results in signal quenching rather than the desired increase in signal intensity.

As the decay characteristics of radioisotopes are pre-defined, enhancing the intensity of a radioactive label can merely be achieved by selecting an isotope that displays ideally adapted properties to the surgical application. Critical parameters in this context are the type and energy of the emitted radiation and potential (unwanted) co-emissions, and the half-life of the radionuclide. In the case of fluorescent dyes, major parameters for their selection are (photo)stability, quantum yield (Q_F_) and brightness (= QF * extinction coefficient (ε), Fig. 5). From translational perspective cyanine dyes seem to be the “best fit” for these prerequisites. Uniquely, these dyes can be chemically modified to increase their brightness. In cyanine dyes, this feature is directly related to the conjugated bond length that in turn dictates the fluorescent wavelength. A prime example herein is the effect that the length of the bridge between the indole units has on the brightness of cyanine dyes (Fig. 5b [76]), . This is also reflected in the relatively poor brightness of the well-known dyes ICG and IRDye800CW [71]. Also the conformational flexibility plays a critical role in cyanine dyes (trans (fluorescent) vs. cis (dark)) [112], with trans-conformational rigid dyes displaying a superior brightness. Various literature reports also indicate that the introduction of -SO_3_^−^ moieties on cyanine dyes enhances their brightness [50, 61].

## Conclusion

In summary, generating clinically useful targeted imaging agents for image-guided surgery involves a multifaceted approach that ultimately aims to enhance surgery. Several interrelated parameters such as receptor affinity, internalization behavior, lipophilicity, net charge and charge distribution, PPB and well as excretion route and kinetics require careful balancing and finetuning. Since high receptor affinity is at the core of effective targeting, the selection of a pharmacophore optimized for receptor interaction is central, but challenges arise when small pharmacophores are combined with large imaging labels. Here, the introduction of spacers and structural optimization help mitigate these challenges. PPB significantly influences in vivo performance, impacting blood retention, biological half-life, and tumor uptake and may be fine-tuned by charge-based modifications. The same toolbox may be applied to adapt the clearance route of an imaging agent for optimal clinical applicability towards enhancement of surgery. Of all parameters, signal intensity deserves particular focus for applications in intraoperative guidance as it can directly influence the surgical decision making. It largely depends on overall imaging agent characteristics and dosing, but also on the relation between receptor expression/binding and non-specific uptake that together determine and the detectability of the imaging label within the surrounding tissue. Thus, ligand design for surgical guidance requires careful balancing of numerous variables, and a comprehensive understanding of these multidirectional optimization strategies is essential for successful development and clinical application of targeted imaging agents in intraoperative molecular imaging.

## Data Availability

This is a literature review, data analysed is included in the present manuscript.
